# Pricing the priceless child 2.0: children as human capital investment

**DOI:** 10.1007/s11186-022-09508-x

**Published:** 2022-12-08

**Authors:** Nina Bandelj, Michelle Spiegel

**Affiliations:** 1 Department of Sociology, University of California, Irvine, CA USA; 2 School of Education, University of California, Irvine, CA USA

**Keywords:** Children, Education, Human capital, Investment, Social value, Viviana Zelizer

## Abstract

This article takes Viviana Zelizer’s (1985) *Pricing the Priceless Child* to the new millennium. Zelizer documented the transformation between the 19th and 20th century from an “economically useful” to an “emotionally priceless” child. She observed that by the 1930s, American children were practically economically worthless but invested with significant emotional value. What has happened to this emotionally priceless child at the dawn of the new millennium? Has there been a new transformation in the social value of children, and, if so, what might have such a transformation entailed? To address these questions, we examine overtime trends that point to increasing devotion of resources and time to children’s education, a key input in the exceedingly influential human capital theory, which connects investment into children’s human capital with their future market value. Therefore, we argue that the priceless child 2.0 is a useful-to-be human capital investment child. We use four empirical examples of overtime growth in children’s human capital investment: (a) enrollments in early childhood education, (b) federal spending on early education, (c) federal spending on K-12 programs, and (d) parental spending on child care, education and extracurricular activities. In the conclusion, we discuss some potential consequences and concerns about raising children as human capital investment.

## Introduction

In 1985, Viviana Zelizer published *Pricing the Priceless Child*, a magisterial work on the changing social value of children in the United States between 1870 and 1930. Zelizer began her analysis in the period when children held notable economic roles as contributors to the family welfare, often working as factory laborers. However, cultural and economic transformations of the early 20th century undermined the importance of children’s economic utility to the household and, instead, led to what Zelizer called sacralization of children as emotionally priceless. Children came to occupy central locations in the emotional life of the family just as they were moved to the periphery of the household’s economic sphere. In Zelizer’s words, “[t]he newly sacred child occupied a special and separate world, regulated by affection and education, not work or profit” (p. 209).

Zelizer’s (1981, 1985) historical analysis ends around the 1930s. Given broad societal changes since that period, we stipulate that the social value of children in the United States has undergone further changes since the mid 20th century. We argue that those changes signal an increasing view of children as useful for their human capital. The concept of human capital was popularized by economist and Nobel Prize Laureate Gary Becker. Since the 1960s it has become contemporary lingua franca and deeply embedded within the public imaginary (Brown, 2015; Zuidhof, 2012). We suggest this concept has also had a profound effect on how the policy makers and social scientists view the contemporary social value of children.

Regrettably, there are but a few empirical studies that provide a window into cultural understandings of children and their social value at the dawn of the 21st century. A rare piece of evidence comes from historian Julia Wrigley’s (1989) analysis of parenting advice published in popular magazines between 1900 and 1985. Wrigley documents a shift from a focus on medical and nutritional advice to an increasing focus, beginning in the 1960s, on the intellectual development of children. According to Wrigley (1989), cognitive and intellectual development of children became a pronounced concern for middle-class parents, as they began to see these traits as crucial for children’s future economic success.

Another important strand of research relevant to our argument is about the changing norms of child rearing, encapsulated by the ideology of intensive mothering. Coined by Hays (1996: p. *x*), intensive mothering describes strong cultural norms for mothers, “to expend a tremendous amount of time, energy and money in raising their children,” from birth through the transition to adulthood. In the past few decades, sociologists have written extensively on not only intensive mothering but intensive parenting (Ishizuka, 2019), including heavy involvement in children’s schooling (Lois, 2012) or health and nutrition (Reich, 2014), as well as the conflicting demands on mothers to spend time raising children and working outside the home (Blair-Loy, 2005; Ennis, 2014; Stone, 2007).

Stimulated by this research, the purpose of the present essay is to take Zelizer’s *Pricing the Priceless Child* to the new millennium. We ask whether there has been a new “profound transformation in the economic and sentimental value of children” (Zelizer, 1985: 3) since the 1980s when the book was originally published. Our analysis also responds to calls to challenge taken-for-granted worldviews that guide contemporary public debates concerning children (Spyrou, 2021). We begin by entertaining Zelizer’s own stipulations about potential new turns in the social value of children, as revealed in her final chapter of *Pricing the Priceless Child*. Zelizer asked in that final chapter how the emotionally priceless but economically useless child may become newly useful. She offered two possibilities. One option was that children could become newly useful in the home, contributing more to household labor. The second alternative for children’s potential return to usefulness identified by Zelizer was that children would increasingly partake in paid work. We present secondary data from child time use studies and labor market participation to evaluate both scenarios and find that evidence does not align with either of them. Instead, and as we elaborate in the second part of the essay, overtime trends point to increasing devotion of resources and time to children’s education - a key input in Becker’s human capital theory - which is purported to make children more economically useful as adults. To develop this argument, we first review the propositions of the human capital theory and its empirical measurements in terms of education, and then proceed to present evidence of overtime trends in children’s preprimary education, education spending, and education-related activities to show increasing societal and parental focus on human capital investment in children. In the conclusion we discuss some potential consequences of raising children as useful for their human capital and point to those who voice concerns about these trends.

## Zelizer on the new potential usefulness of children

In the last chapter of *Pricing the Priceless Child* titled “From Useful to Useless and Back to Useful? Emerging Patterns in the Valuation of Children,” Zelizer speculates about new potential changes in the valuation of children at the time of the book’s completion in the early 1980s. She wonders whether “in the 1980s, the sacred, economically useless child may have become a luxury or an indulgence that the contemporary family no longer values, nor in fact, can afford” (p. 208).

The quote with which Zelizer begins her last chapter is telling. She takes from Sarane Boocock’s (1976) essay in which Boocock states, “Although one would not wish to return to an era of exploitative child labor… one still has the feeling that children in societies like ours are underemployed” (p. 208). This serves as a springboard for Zelizer to offer potential ways in which children can become newly useful, either through their participation in the household division of labor, or through “innovative ways to include children in the productive life of the community” (1985: 209, cf. Zelizer, 2002, 2005, 2012).

### Children’s usefulness in housework contributions

First, let’s interrogate the possibility that Zelizer entertains about the new emergence of a valuable “housechild.” How much have children contributed to the household since the 1980s? Have there been increasing or decreasing trends? To be sure, it is hard to generalize to all children. For instance, research points to the productive role especially of immigrant children, who often serve as interpreters to the family and as supporters with schoolwork and homework for younger siblings (Lanuza & Bandelj, 2015; Orellana et al., 2003). Further, scholars have drawn attention to the everyday caregiving roles of children (Marschall, 2014; Souralová, 2017) and during the COVID-19 pandemic (Balagopalan, 2021).

Still, what may have been some broad stroke trends across a representative sample of American children, and over time? Here, nationally representative time diary studies are helpful. Using data from the Panel Study of Income Dynamics, Hofferth and Sandberg (2001) compare the period between 1981 and 1997 to find that the proportion of children who mentioned, among their weekly activities, doing basic household tasks such as cooking and cleaning has declined by about 22% during this period (Hofferth & Sandberg, 2001). While in 1981, 92% of children ages 9–12 mentioned chores among the weekly activity they participated in, this number was down to 72% by 1997. Between 1997 and 2003, a further nearly 9% decrease was observed in the proportion of children ages 9–12 who said they participated in daily household work (Hofferth, 2009). These statistics shed doubt that, since the 1980s, the new utility of children would come from their increased contributions to household labor. Interestingly, an American Psychological Association podcast recorded in July 2017 discussed a study which found that “82% of adults reported doing chores as a child but only 28% were having their own children do household chores” (Arsenault, 2017). Moreover, an ethnography and interview-based study on children’s chores revealed that “most children spend surprisingly little time helping around the house and engage in fewer tasks than what they report in interviews” (Klein et al., 2004: 98). One then wonders, how do children spend their time, if they are not contributing to household chores? Given the broader societal emphasis on work and rise of ideal worker norms (Beckman & Mazmanian, 2021), do they devote more time to paid work?

### Children’s involvement in paid work

The second alternative that would increase the usefulness of the emotionally priceless child, which Zelizer envisioned in the early 1980s, was to see children as active participants in some aspects of paid work. Indeed, Zelizer cites fears from observers that “economic dependency can be a psychological hazard to children” (p. 220). She references work of psychologist Mary Engel and collaborators (1967) who found “that part-time jobs not only had no negative effects among boys between ten and fourteen years of age but helped them in feelings of competence and personality development” (p. 220). Zelizer concludes her book, stipulating: “Perhaps, within the household, with proper guidance, new attitudes, and safeguards to prevent their exploitation, children may well become invaluable useful participants in a cooperative family unit” (p. 228).

What evidence do we have about how much children actually engage in paid work in the United States, and what have the trends been over time? We consult here again Sandra Hofferth’s (2009) studies of the data from the Panel Study of Income Dynamics, which asked whether market work was among the weekly activities that children ages 9 to 12 engaged in. These analyses showed that a minority, but a non-negligible share, 12%, reported market work as an activity they engaged in a typical week in 1981. However, this proportion significantly declined over time. It was at a low 3% in 1997 and was reduced effectively to zero by 2003.

Moreover, the decline in time children aged 9 to 12 spend in paid work is consistent with a broader downward trend in teen labor force participation. Looking at data from the U.S. Bureau of Labor Statistics, Current Population Survey, labor force participation amongst teens aged 16 to 19 years peaked in 1979, with 57.9% of teens in the labor market. The 2000s saw the greatest decrease in teen labor market participation, with 52% participating in 2000 and just 34.3% participating by 2015. The Bureau of Labor Statistics projects only a 24.6% teen labor force participation rate by 2024. Perhaps more starkly, looking at labor market participation in the summer, when labor market participation was historically highest amongst teens, we see similar substantial decreases. While some 63% of teens participated in the labor market during the summer of 1950, only 43% were participating in the summer labor market by 2016 (Morisi, 2017).

So, what do children and teenagers do if they don’t work? When asked for the major reason for their non-involvement in the labor force, an overwhelming majority, 92% in 2014, cited “going to school” (Hipple, 2015), which has extended into the summer. As Morisi (2017) reports, in 1985, 10.4% of teens were enrolled in school in July, whereas in 2016, this number rose substantially to 42%. In addition, research shows that study time outside of school for children ages 6 to 8 grew from approximately 45 min per week in 1981 to about two hours per week in 1997 (Hofferth & Sandberg, 2001, Schaub, 2010), which likely includes time devoted to homework. Taking from these trends, we will argue that it is a focus on building human capital of children through education and schooling that may have come to constitute their new usefulness, rather than housework or paid work.

In the past few decades, there has been a significant rise in educational investment in children. Economists frame this effort as human capital development, whereby, according to human capital theory, education increases future productivity and wage returns in adulthood. As such, pricing the priceless child 2.0 might mean building children’s human capital. To develop our argument, we will first describe the theory of human capital and its popularization and then show empirical evidence from the United States, across four different domains of public and private educational investment, which is consistent with the argument that contemporary children’s social value is increasingly placed on their human capital.

## The prominence of human capital

While human capital theory was developed starting in the 1960s, its prominence grew after Zelizer’s (1985) publication of *Pricing the Priceless Child*. In this section, we describe key features of human capital theory, and its measurement, that are relevant to understanding changes in the social value of children.

### Gary Becker and human capital theory

While the earliest form of the term “human capital” can be traced to the 1890s, the theory became more developed and primarily associated with economist Gary Becker in the 1960s (Becker, 1964; Kiker, 1966). Becker cites his interest in developing the theory as his contribution to “the age-old quest for an understanding of the personal distribution of income” that physical capital or labor growth could not explain (1993: 12). To do so, Becker turns to resources or assets embodied within individuals. Prior to Becker, the public was resistant to conceptualize people as marketable assets (Becker, 1993; Schultz, 1961). Over time, and for a variety of reasons, human capital theory has become lingua franca (Brown, 2015) such that human capital theory as a style of economic reasoning now forms the implicit foundation of policy formation (Allison, 2006; Berman, 2022; Hirschman & Berman, 2014).

Becker defines the concept in his book *Human Capital* quite simply as “activities in the present [that] affect future well-being” (Becker, 1964). He writes, “investments in human capital [are] activities that influence future monetary and psychic income by increasing the resources in people” (Becker, 1993: 11).^1^ The “activities in the present” that build human capital for Becker in his original writing primarily include education (Teixeira, 2014), which is conceptualized as an economically productive system (Griffen & Panofsky, 2021). From this perspective, framing education processes as “investments in human capital” is a case of “economization” whereby phenomena traditionally outside of the domain of neo-classical economics are transformed into economic problems and subject to economic reasoning (Griffen & Panofsky, 2021: 515; cf. Çalişkan & Callon, 2009).

Becker’s work on human capital received great prominence and was central to his recognition as the Nobel Prize Laureate in 1992. As the description on the flap of *Human Capital* book’s third edition states, “Becker’s research on human capital was considered by the Nobel committee to be his most noteworthy contribution to economics.” Becker himself describes the influence of his research, some 40 years after the book’s original publication, writing that the human capital theory “mushroomed throughout the world and stimulated a profusion of research and policy proposals” (p. xxi, 1993). In the introduction to the second edition three years later he writes, “the mushrooming has continued unabated” (p. 3, 1996). In fact, observers note that the notion of human capital is in many ways a default logic and deeply embedded within the public imaginary (Brown, 2015).

### Key assumptions and measurements of human capital theory

A few key assumptions of human capital theory are important for understanding the theory’s impact on how we envision children’s contemporary social value. First, the theory was an intervention in a broad academic debate attempting to explain macroeconomic phenomena like economic growth and the income distribution in a society, as a function of, and through the characteristics of, individuals (Goldin, 2016). The explanation of national growth and even social mobility as a product of realized capital embodied in humans (Marginson, 2019) provided a moral and economic justification for public and private investments in education to enhance human capital. Importantly, modifications or augmentations to the resources within a person, human capital, has become linked directly to increases in future monetary income.^2^

Further, “activities in the present” (Becker, 1993: 11) that would constitute human capital are amenable to quantification, and thus measurement. In sociology, top cited articles in the *American Sociological Review*, the discipline’s premier journal, with the phrase “human capital” in the title, operationalize human capital in broadly similar ways. For example, Coleman (1988) uses dropping out of high school as a measure of human capital (or lack thereof). Others use English language skills, education levels completed, or years in the labor market as measures of one’s human capital (Sanders & Nee, 1996; Zhou & Logan, 1989). In psychology, most articles invoking human capital are published in business and management journals (see Coff and Kryscynski, 2011; Crook et al., 2011; Luthans & Youssef, 2004). These studies operationalize human capital similarly to sociology studies referenced earlier, namely by using educational attainment as well as measures of technical training and work experience (Johnson, Schnatterly, and Hill, 2013; Ployhart et al., 2011). Further, examples of how human capital is measured in recent economic literature include secondary educational attainment (Marx & Turner, 2018), students’ grade point averages (Lyle, 2009), and work experience (Bartel et al., 2014).

A strand of economics research that is particularly relevant to children relies on psychometric tools to measure human capital. Beginning in the 1950s with the evaluation of early childhood programs (including Abecedarian, Perry Preschool, and the Head Start Impact Study), this literature has focused on skill development – conceived as human capital development - before children enter school (Campbell et al., 2012; Weikart, 1967). Psychometric tools are used to measure changes in human capital resulting from early childhood interventions, including scales like the Peabody Picture Vocabulary test. For instance, this test is used recently by Attansio and colleagues (2020) who also describe the quality of parents’ interactions with children as linked to enhancing (or not) children’s human capital. There is also evidence that early childhood analysts have not lost sight of the human capital concept’s economics origins. This is revealed in Cunha, Nielsen, and Williams (2021) where authors suggest that a major problem with relying on psychometric scales to measure human capital is that the different values in the scale have no inherent economic meaning. To resolve this, they suggest correlating scale results to economic outcomes, such as future wages, to give economic meaning to changes in psychometric scale values.

The reviewed scholarly literature in sociology, psychology and economics that uses and measures human capital as education and skills linked to future productivity and wages shows pointedly how human capital theory would shape our understanding of children’s usefulness, namely, that children’s future economic value would result from human capital investment during childhood. This literature, as well as Gary Becker’s initial formulations of inputs to human capital, also offers suggestions of where to look for empirical evidence of our hypothesized growing focus on investment into building children’s human capital over time, particularly as it relates to investments in education in both public (by government) and private (by parents) domains.

## Overtime trends in children’s human capital investment

To document the transformation from an economically useful to an emotionally priceless child, Zelizer (1985) examined the historical development of three monetary processes, including child life insurance, compensation for the wrongful death of children, and the sale of children through adoption. In all these cases, monetary payments had very strong symbolic meanings: “An insurance policy, for instance, never sold as a sensible investment but as a token of respect for the dying child in the nineteenth century, and later as a token of love for the living child” (p. 211). Zelizer investigated “wrongful death awards, adoption, and insurance markets” because they revealed something about “cultural definitions of childhood” (p. 212).

Likewise, we will document monetary processes that would reveal a potential new cultural definition of childhood that we expect has taken hold, the one related to increasing focus on building children’s human capital. Specifically, we will show the overtime trends in (a) enrollments in early childhood education, (b) federal spending on early education, (c) federal spending on K-12 programs, as well as (d) parental spending on child care, education and extracurricular activities. Focusing on these four examples we don’t mean to suggest that these are the only important processes that have taken hold related to children. Nevertheless, following Zelizer, we consider them revelatory of the social meaning of money whereby monetary spending indicates investment into outcomes deemed valuable by society, in our case, prizing human capital formation in children.

### Enrollment in educational programs before primary school

In the first half of the 20th century, children typically did not begin formal schooling until age 5 or 6 (Vinovskis, 1993). While there were efforts to create infant schools dating back to the mid-1800s, parents were mostly encouraged to provide “adequate indoor playing space for their children” (Zelizer, 1985, p. 51). Yet, in the 1960s, this began to change. Around the time Becker (1964) published his first book on human capital, the Current Population Survey of the U.S. Census began to collect data on preprimary school enrollment. We use this historical data, beginning when the information started to be formally collected, to demonstrate the growing prevalence of formal education in children’s lives over the past 50 years.

**Fig. 1:**
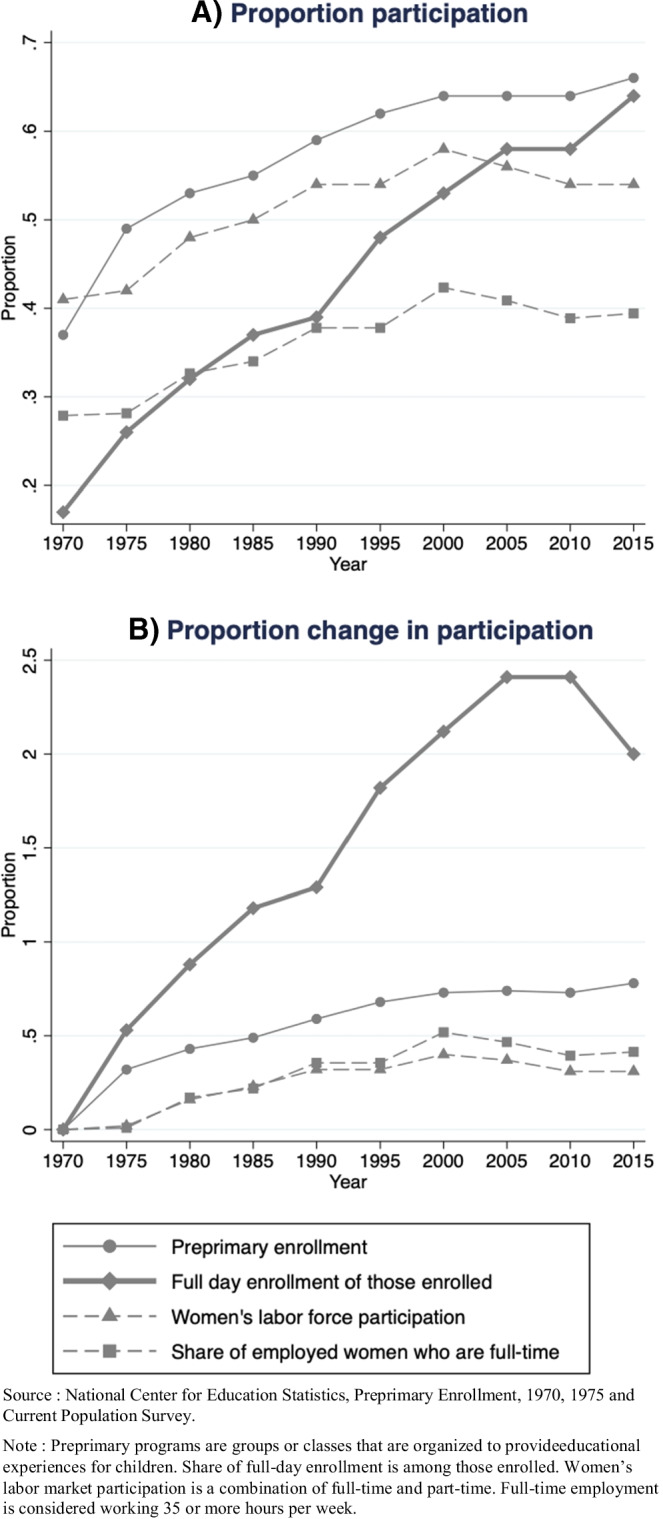
Children’s enrollment in preprimary programs, 1970–2015

In Panel A of Fig. 1, the two solid lines show the proportion of children enrolled in preprimary programs, defined as “a group or class that is organized to provide educational experiences for children during the year or years preceding kindergarten” between 1970 and 2015 (U.S. Census, 2019). Looking at the solid line with the circle marker, we see that in 1970, 37% of American children aged 3 to 5 were enrolled in formal education at the preprimary level. The percent of children enrolled in preprimary education increases overtime, with about 64% of children aged 3 to 5 in formal schooling by 2015. Further, as shown by the solid line with the diamond marker, children are spending larger shares of their day in preprimary education. While in 1970 only 17% of children in preprimary programs were enrolled in full-day programming, some 64% of those enrolled were spending their whole days in preprimary education by 2015.

Admittedly, the 1970s is also a period of women’s entry into the labor market, which undoubtedly influences children’s environments. As shown in the dashed line with triangle marker in Fig. 1, Panel A, between 1970 and 2015, women’s labor force participation increased from 41% to 54% (U.S. Bureau of Labor Statistics, n.d.; U.S. Census Bureau, n.d.). In the dashed line with square markers, we see that among employed women, women’s full-time labor force participation mostly stayed constant. As primary caregivers were leaving the home, there was a need to organize children’s time and space. However, the rate of the increase in children’s enrollment in preprimary programs since 1970 exceeds the rate of the increase in women’s labor force participation. As shown in Panel B, we see that the rate of increase in preprimary enrollment and full-day preprimary program enrollment (solid lines with circle and diamond markers, respectively) is greater than the rate at which women entered the labor market and the rate of change of women’s full-time labor market participation (dashed lines with triangle and square markers, respectively). Thus, we propose that the rise in primary caregivers’ growing participation in the labor market alone cannot be solely responsible for increase in children’s preprimary enrollment. Instead, we posit that the growth in preprimary enrollment, and especially the noticeable growth in full time enrollment since the mid 1990s (solid line with dashed marker), at which point women’s labor force participation levels off (dashed line with triangle marker), demonstrates the rising importance of investment in children’s human capital that would render children as economic assets, only not in the present as Zelizer’s analysis of laboring children in the 1900s revealed but, rather, in the future when they become adults and their accumulated human capital can influence the price they can command in the labor market.

### Federal spending on early education: funding for Head start

**Fig. 2:**
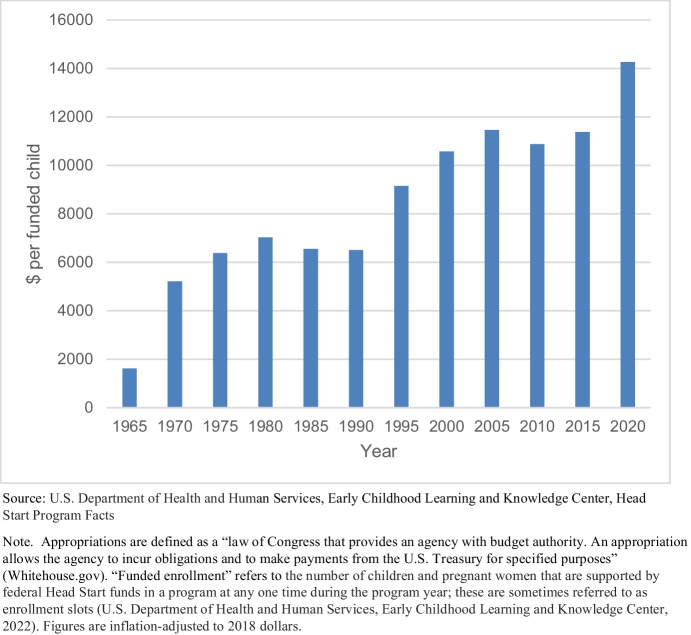
Head Start appropriations, 1965–2020

As data presented in Fig. 1 showed, an increasing share of children in the United States are spending their early years in preprimary education programs and many are in full-day preprimary education programs. Of these programs, one federally funded one holds national prominence: Head Start. Founded in 1965, Head Start formed a part of President Lyndon B. Johnson’s Economic Opportunity Act (EOA), known as the War on Poverty. Since its founding, appropriations earmarked for Head Start have consistently grown, which we take to reflect commitment to devote public resources for children’s preprimary education.^3^ Figure 2 shows the federal appropriations for Head Start (in 2018 dollars) per enrollment slot^4^ between its official founding in 1965 and 2020. In 1965, federal appropriations totaled near $1,616 per funded child (in 2018 dollars) and have grown ever since. Appropriations reached $6,550 per child in 1985, almost $11,500 in 2005, and more than $14,000 in 2020.

While some envisioned that Head Start would address not only children’s academic skills but also their health, their family’s well-being, and racial justice, we glean from the leadership of the panel of experts the increasing emphasis on the program’s cognitive development effects on children (Sargent Shriver, 1964). Further, the emergence of the notion of a “critical period” of early childhood – a stage of development during which neuroscientists have found children’s brains to be more malleable – as a focus of economists’ research program on human capital development has contributed to policy changes in the past fifty years, emphasizing the role of Head Start in building children’s academic progress and skill-attainment in language, literacy, and mathematics (Head Start Act, 2007; Paulsell et al., 2006; Griffen, 2022a).^5^ Indeed, it is research on building children’s human capital that continues to inspire federal funding proposals targeting public preprimary programs (The White House, 2021). Thus, the trend of increasing funding for Head Start over the past 50 years suggests that investment through this program is viewed as useful for children’s human capital development.

### Federal spending on K-12

Like spending on preprimary education, spending on primary and secondary education has increased since the 1960s as well. To illustrate this, we draw on data from Urban Institute’s reports on federal spending on children (defined as residents of the United States under age 19) (Carasso et al., 2007; Hahn et al., 2021). These reports classify about 100 federal programs^6^ into major budget categories.^7^ We include here the categories of tax credits and exemptions (tax spending for short), income security, and education. Programs in the tax spending category^8^ include, for example, the Earned Income Tax Credit and the Child and Dependent Care Tax Credit; the income security category includes spending on programs such as Social Security and Temporary Assistance for Needy Families (TANF); and the education category includes funding for programs like Impact Aid and Education for the Disadvantaged and excludes any spending or tax programs that finance post-secondary education.^9^

**Fig. 3:**
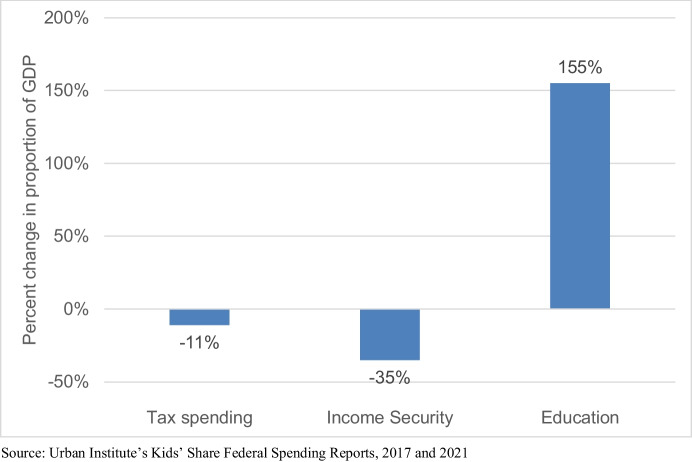
Change in federal spending on children, 1960–2020

Figure 3 shows changes in the proportion of GDP allocated towards the three budget categories. Between 1960 and 2017, the proportion of GDP spent on tax credits and exemptions and income security programs has decreased while spending on education as a proportion of GDP has substantially increased. Specifically, tax spending has decreased by 11%, constituting 1.28% of GDP in 1960 and 1.14% of GDP in 2020^10^, and income security spending has decreased by 35%, constituting 0.43% of GDP in 1960 and 0.28% in 2020.^11^ The opposite trend is true for education. Some 0.09% of GDP was allocated towards education in 1960. This increases to 0.23% of GDP in 2020, which is a 156% increase from 1960, as depicted in Fig. 3.^12^ We note that public resources devoted to children in the United States may be overall lower than in comparable advanced industrial countries, and there may be great disparities across individual states and counties. Nevertheless, we believe it is telling where the largest share of growth (if there is one) is happening: in funds earmarked for children’s education. While the largest and most influential federal legislation on education spending – the Elementary and Secondary Education Act (ESEA) passed in 1965 – was formally justified through a language of equality, as time passed, spending on education became about improving efficiency for building children’s human capital (Griffen, 2022b).

Astute policy observers will note that most of the education spending is sourced from state and local levels and may be curious as to whether these trends are offset by decreasing spending at the state level. We find this not to be the case. Using state spending data from the Urban Institute available from 1997 to 2017, we find that spending on education at the state level has increased. U.S. states spent on average 150% more on K-12 education in 2017 as they did in 1997.^13^ Thus, spending at both the state and federal levels on K through 12 education has increased. We take these trends to suggest that public resources towards children are valuable insofar as they are directed towards building their human capital through K-12 education. Importantly, we make no claims that these federal budgetary trends suggest sufficiency or equality in distribution of resources needed to provide quality public education to individual children, or equality of educational opportunity for all racial groups, which remain persistent problems in the United States.

### Parental spending on children’s education

**Fig. 4:**
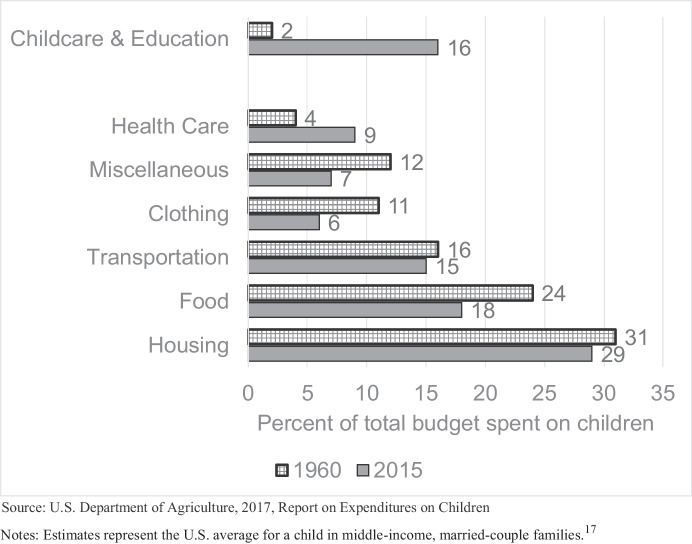
Parental expenditures on a child from birth through age 17, 1960 versus 2015

In addition to larger shares of GDP allocated towards K-12 education and increasing spending on preprimary education over the past 50 years, we also note increases in average parental spending on education-related activities for children over the past several decades. Data from the U.S. Department of Agriculture provides estimates of child-rearing expenditures in 1960 and 2015 for a middle-income, married-couple family (Lino et al., 2017). In Fig. 4, we present shares of the family’s total budget spent on children devoted to a variety of categories, as defined by the Department of Agriculture. In 1960, average expenditures on a child within a married-couple family with middle-income level totaled to $25,229 (Lino et al., 2017: 21), which amounts to $202,020 in 2015 dollars. As shown in Fig. 4, only 2% of the total expenditures of the 1960 household went toward childcare and education.^14^ Conversely, by 2015, estimated expenditures on raising a child to age 18 amounted to $233,610 in 2015 dollars, of which 16% was going towards childcare and education, as shown in Fig. 4. This means that the share of a family’s total budget going towards childcare and education category increased eight-fold between 1960 and 2015. Figure 4 shows that only one other category of spending saw an increased share of the total budget devoted to it, and that is health care costs, but the increase was not nearly as substantial as that for the childcare and education category.

The increased expenditures going toward children’s education-related activities is also reflected in analyses presented by demographers Kornrich and Furstenberg (2013) using the Consumer Expenditure Survey data. The authors find that in 1972-73 parents of children ages 0 to 24 expended $621 dollars on average for children’s education, including “room and board at school; tuition, fees, and books; private recreational lessons; and other educational expenses” (p. 8). This amount grew to $1,189 per year in 2006-07, a more than 90% increase. For comparison, spending on accessories (including books, toys, games, and clothing) for children decreased during this same period from $513 to $463. We take these trends to suggest that parents are increasingly devoting resources, and a bigger share of resources, to education of their children.

This said, there certainly exist social class gaps in parental spending on children (Kaushal et al., 2011; Schneider et al., 2018). Nevertheless, we also have evidence that enrichment expenditures on children have increased across both bottom and top income quintiles between 1972 and 2006 (Duncan & Murnane, 2011, p. 11) and that the share of spending on childcare and education as a proportion of all parental spending has increased for families in the bottom, middle and top third of the income distribution between 1995 and 2015 (Lino et al. 2017). This suggests that the focus on educational investment is observed across American families, even if more or less pronounced across different social classes.

**Fig. 5:**
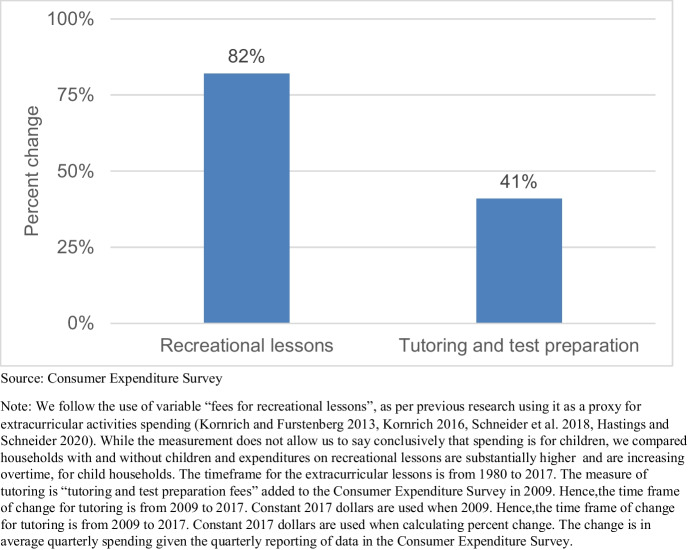
Change in parental spending per child on extracurricular activities

As a final example, we illustrate changes in parental spending on extracurricular activities for children overtime. Researchers have noted that parents are organizing their children’s time increasingly around activities outside of school (Lareau, 2003; Levey-Friedman, 2013, Ishizuka, 2019, Dhingra, 2020). While parents may have multiple motivations to enroll children in extracurricular activities (Dhingra, 2020), several scholars have argued that enrichment activities have a direct impact on cognitive development and various skills like confidence and self-discipline that may positively impact children’s future economic outcomes (Duncan & Murnane, 2011; Kaushal et al., 2011). Overtime data on extracurricular activities are scarce. We follow previous research and rely on an indicator from the Consumer Expenditure Survey frequently used in studies of parental investment (Kornrich & Furstenberg, 2013, Kornrich, 2016, Schneider et al., 2018, Hastings & Schneider, 2021), “fees for recreational lessons,” and depict a change in average quarterly spending per child on recreational lessons and fees from 1980 to 2017. In addition, we note that in 2009 the Consumer Expenditure survey added a new category of spending focused on tutoring, which we also take as a proxy of increasing focus on human capital investment. As Fig. 5 shows, average quarterly spending per child in households with children increased for 82% between 1980 and 2017 for recreational lessons expenses and 41% between 2009 and 2017 for tutoring expenses.

## Discussion

To continue Viviana Zelizer’s (1985) analysis, we have taken the priceless child to the 21st century and identify a new stage of the transformation in the social value of children. We used evidence of monetary spending and investment by the state and by parents for children’s education to argue that the priceless child 2.0 has yet again become economically useful, just not in the present by contributing labor as children did in the 19th century. Rather, the 21st century child is construed to be economically useful because she is invested with human capital that would increase her economic value as an adult in the future labor market.

Gary Becker became famous and lauded by the Nobel Prize committee for introducing the economic style of reasoning to traditionally non-economic spheres of life. The spread of economic style of reasoning has propelled the specific ideas of human capital investment into public discourse and public policy (Brown, 2015). Human capital theory can be understood as a critical intervention in the drastic reorganization of children’s time and space that put children’s future economic value front and center in a process that we could call *human capitalization*. We see this human capitalization as but one case study of processes of economization that others have documented in other spheres of social life (Berman, 2014; Çalişkan & Callon, 2009; Griffen & Panofsky, 2021; Spring, 2015).

Indeed, there is evidence to suggest that centrality of educational investment for children’s future labor market value is not unique to the United States. As Wotipka et al. (2017) observe, “the world average gross enrollment ratio in ECCE [(early childhood care and education)] was less than 10% in 1965 (Inkeles & Sirowy, 1983), but it had increased to more than 64% by 2010 (UNESCO, 2006)” (p. 307). The authors continue by noting that this trend is not simply about providing care for children outside of the home, but that “international events repeatedly emphasiz[ed] the significance of ECCE as an investment in future human capital and a right of children (UNESCO, 2006)” (p. 309). To be sure, state investment in children structures parental investment in children – and inequalities thereof. Kornrich et al. (2020) write that “private spending on care for children and children’s education is more unequal in the United States than in Norway, Australia, or Spain” (p. 581) and attribute this to the lower level of public provision of education and childcare in the United States compared to the other countries. However, the point remains that children’s time and space are likely being substantially reorganized across many of the world’s countries, with an emphasis on shaping children’s human capital.

While our evidence of increasing investment in children’s formal education suggests centrality of human capital formation, the extent to which the ultimate goal of education should *indeed* be boosting of human capital is not uncontroversial. Labaree (1997) posits that there have been three conflicting goals for K-12 education overtime, including democratic equality, social efficiency, and social mobility. The democratic equality goal claims that education should prepare citizens, while the social efficiency goal claims that schools should focus on training workers. The social mobility goal claims that schools should prepare individuals to compete for social positions. Labaree (2011) expressed that one goal has won out over the others, writing that “the economic rationale for schooling in the United States has gradually grown in intensity, and in the twentieth century it became increasingly explicit as a primary goal for education” (p. 381). The social efficiency and social mobility goals speak to human capital concerns, but democratic equality adds the grave importance of preparing young people to develop a sense of collective responsibility as citizens in a democratic society. From this, one can speculate how privileging human capital concerns in education may be done at the expense of building values of democratic equality.

Moreover, the extent to which investment in formal schooling actually boosts skills and productivity is also put into question. Social critics as well as some economists have questioned the extent to which more education increases human capital or merely signals credentials (Caplan, 2018). Some sociologists have posited that these trends in rising educational attendance are more related to growth in educational credentialism (Brown, 2001) that maintains structures of socioeconomic hierarchy. In addition, some research finds that parents themselves have multiple motivations for spending on their children that are not explicitly human capital oriented (Gauthier & de Jong, 2021; Zaloom, 2019). Indeed, and importantly, our argument about increasing human capitalization of children does not assume that this is necessarily the guiding principle of individual parents in how they treat their children. Rather, we focused in this analysis on the government’s actions, policies and social scientific expertise in shaping a specific contemporary cultural understanding of a social value of children. It remains an empirical question, to what extent individual parents align with this human capital logic, and possibly the differences across class/race/ethnicity in the extent this logic is embraced.^15^

The centrality of human capital investment is also a political issue. In a 2008 speech, President George Bush emphasized that “strengthening education systems” is how we can unlock “the greatest resource” of society for economic growth - “the skills and talents of the people. Or… human capital” (Bush, 2008). This human capital focused understanding of the role of education cuts across the political aisle. President Obama, too, promoted early childhood education as a means for improving economic efficiency and reducing inequality (Dillon, 2008). When speaking on a policy to improve the quality of Head Start, President Obama said that in a time when a company is able to move wherever it wants and “will make that decision based on where they can find the most highly skilled workforce, it is absolutely imperative that we make sure that the United States is the place where we’ve got the best trained, best-educated young people…it’s an economic imperative” (Obama, 2011). For many, such thinking makes perfect sense as a natural course of action. In contrast, we suggest that there is little that is natural about these claims. Instead, human capital investment has been socially constructed as a value, which we see linked to the rise in prominence of the economic style of reasoning (Berman, 2022) that, we argue, has influenced not only public policy but also how we think of children and what we should do for them.

## Conclusion

The goal of this paper was to take inspiration from Viviana Zelizer’s (1985) trailblazing study of the changing social value of children in the United States to examine how this value may have changed again since Zelizer’s analysis. Zelizer documented the transformation from what she called an economically useful to an emotionally priceless child. She observed that by the 1930s children were practically economically worthless but invested with significant emotional value, a trend that began with the middle and upper classes but soon got absorbed by lower class families as well. We asked: What has happened to this emotionally priceless child in the 21st century? As Zelizer was finishing her book in the early 1980s, she speculated that perhaps the era would see a return of a useful child, perhaps one that contributes more to the household economy, as their mother goes to work outside the home, or possibly even one that participates more in paid work. As we show in the first part of this article, neither of these two potentialities materialized. Quite to the contrary, children’s participation in household chores and in market work has markedly declined since the 1970s. Is the contemporary child then just a completely economically useless emotionally priceless creature?

We argued, no. We proposed that the new utility of a late 20th and 21st century child has come to be understood as their *future economic value*, in particular the price they will be able to command on the labor market if they are invested with requisite education and skills, or what many social scientists now understand as human capital. We suggested that the rise of the human capital theory, part and parcel of broader processes of economization, has created a new imaginary of an *economically useful-to-be child* that is entwined thoroughly with economic ideas of human capital investment. Specifically, we delineated the rise of human capital theory, as espoused by economist Gary Becker, and how it has influenced spheres of public policy decision making, including those related to investment in children. We provided four empirical examples of growth in human capital investment in children, including trends in (a) enrollments in preprimary education, (b) federal spending on early education, (c) federal spending on K-12 education, and (d) parental spending on child care, education and extracurricular activities. These all pointed to significant overtime growth in investment in children’s human capital that would, or so the theory predicts, shape children’s future usefulness and productivity. In brief, we argued that the priceless child 2.0 is a human capital investment child.

We also had a unique opportunity to ask Professor Zelizer to comment on the potential recent transformation in the social value of children that may have taken place after her analysis in *Pricing the Priceless Child*. According to Zelizer,

One can detect early glimmers of the 21st century “human capital investment child”. By the 1940s, for instance, children’s life insurance policies, initially sold as burial insurance for poor children, were now being touted as “nest eggs” creating funds for their education. Even earlier, an adoptive couple was reported applying to the New York State Charities Association for a three-month-old baby “who could eventually go to Princeton.” Jumping forward to the recent pandemic, parental concern with their children’s education tangibly impacted household economies. With stay-at-home orders and online schooling, mothers (more often than fathers) took over home schooling responsibilities, sometimes at the expense of keeping their paid employment.”^16^

We hope to inspire other researchers to elaborate on our thinking and present further evidence about, or against, the centrality of human capital investment in how social scientists and policy makers, as well as parents, think about children. We also hope to stimulate studies to better understand the consequences of these human capitalization processes. Some voices of concern already exist. For instance, political theorist Wendy Brown makes the case that thinking of education “as primarily valuable to human capital development, where human capital is what the individual, the business world, and the state seek to enhance in order to maximize competitiveness… undermines democracy itself” (Brown, 2015: 176). Along these lines, feminist scholar bell hooks (1994: 18–19) conceives of education “as the practice of freedom…. that connects the will to know with the will to become.” Others opining on early childhood education curricula argue for alternative formats of schooling that do not emphasize cognitive skills or the ability to execute self-control and prioritize executive function. Rather, they argue for the value of child-centered curricula that bolster creativity and a sense of oneself as part of a greater collective, not in competition with others (Kessler & Swadener, 1992). Yet others point to incompatibility of claims on behalf of children with economistic cost benefit analysis: “Above all, the claims on behalf of children have always been moral ones, and moral positions of all kinds have never had a place within benefit-cost analysis. The utilitarian approach may help children for the moment, but over the long run it will neglect those moral principles and non-instrumental goals that have always been at the center of claims for children” (Grubb & Lazerson, 1988: 324). Clearly, controversies and polemics related to how we envision children will continue. But scholars’ analyses of these issues, as was ours, will remain forever guided by Viviana Zelizer’s path-breaking work that brought to the fore, so compellingly and demonstrably, the moral, not essential, nature of the value of children.

## Footnotes

1 The second part of Becker’s definition of human capital points to “future well-being”. In his original text, he defines future well-being as “monetary income or psychic income” (clarifying that psychic income is another way of saying consumption, 1993, p. 11). The range of “activities in the present” that can be considered as “investments in human capital” have broadened overtime as has the meaning of “future well-being,” encompassing “a person’s appreciation of literature over much of his or her lifetime” (p. 16) as explicated by Becker in 1993. “Investments in human capital” are defined as such because they “yield income or other useful outputs over a long period of time” (p. 15, 1993). While the meaning of “useful outputs over a long period of time” is interesting to explore, it is the “activities in the present” that are demonstrated as shaping children’s environments in the current piece. We speculate on the “useful outputs over a long period of time” in the discussion.

2 Developments in psychology bolstered the idea that resources within people can change in a short time span, and that these changes could be profound in the early years of a person’s life. For example, scholars such as J. McVicker Hunt and Benjamin Bloom argued in the 1960s that children’s intelligence could change due to environmental circumstances, and that the early years of life are a “critical period” (Hunt, 1961). This helped provide the scientific justification for foundational elements of Becker’s human capital theory.

3 Appropriations are laws of Congress that provide an agency with budgetary authority to incur obligations and to make payments from the U.S. Treasury for specified purposes (whitehouse.gov).

4 Enrollment slots are also called “funded enrollment”, which refers to the number of children and pregnant women that are supported by federal Head Start funds in a program at any one time during the program year (U.S. Department of Health and Human Services, Early Childhood Learning and Knowledge Center, 2022).

5 Examples of these policies include the Head Start National Reporting System implemented in 2003 and Improving Head Start for School Readiness Act of 2007.

6 Program inclusion criteria include that (1) the benefits go entirely to children (e.g. the Child Tax Credit); (2) the benefit level increases with the inclusion of children in the application for the benefit (e.g. Medicaid); or (3) children are necessary to qualify for the benefit (e.g. Head Start). For a complete description of the methodology, see Carasso et al. (2007) and Hahn et al., (2021).

7 The list of federal programs counted in the income security category varies slightly by report. Both the 2007 and the 2021 report include: Social Security, Temporary Assistance for Needy Families (TANF) — formerly Aid to Families with Dependent Children (AFDC), Child Support Enforcement, Supplemental Security Income (SSI), Veterans Benefits, and Railroad Retirement. Two additional programs were counted in the 2007 report: Emergency Assistance and Black Lung Disability.

8 The full list of federal programs counted in the tax credits and exemptions category varies slightly by report. Both the 2007 and the 2021 report include: Earned Income Tax Credit (EITC - refundable and nonrefundable), Child and Dependent Care Tax Credit, Child Tax Credit (refundable and nonrefundable), Dependent Exemption, Employer Provided Child Care Exclusion, Employer-Provided Child Care Credit. The 2021 report includes 6 additional programs: assistance for adopted foster children, adoption credit, pandemic response stimulus, qualified zone academy bonds, qualified school construction bonds, and the Premium tax credit.

9 The full list of federal programs counted in the education category include: the Educationally Deprived/Economic Opportunity, Supporting Services, Dependents’ Schools Abroad, Public Lands Revenue for Schools, Assistance in Special Areas, Other, Impact Aid, Vocational (and Adult) Education for the Disadvantaged—formerly Grants for the Disadvantaged, School Improvement, Indian Education, English Language Acquisition— formerly Bilingual and Immigrant Education, Special Education—formerly Education for the Handicapped, Emergency School Assistance (Civil Rights), Education Reform: Goals 2000, Domestic Schools, Reading Excellence, American Printing House for the Blind, Gallaudet University (elementary and secondary schooling), Institute for Education Sciences—formerly Education Research, Innovation and Improvement, Safe Schools and Citizenship Education.

10 However, the proportion of GDP spent on tax exemptions decreased by around 30% between 1960 and 2006 (constituting 0.88% of GDP in 2006). The increase in the proportion of GDP spent on tax credits and exemptions between 2006 and 2020 is largely attributable to tax credits associated with the COVID-19 pandemic.

11 We note that while federal spending on tax credits has declined as a share of total GDP between 1960 and 2020, raw state spending on tax credits has significantly increased between 1997 and 2016 (author calculations; Hahn, Lou, Isaacs, Lauderback, Daly and Steuerle, 2020). Yet, some 25 states do not have their own EITC policies. The Federal EITC is in addition to the state EITC (Center on Budget and Policy Priorities, 2019).

12 We want to note that while education spending as a proportion of GDP has increased between 1960 and 2017, the proportion spent on education remains substantially smaller than the proportion spent on defense, for instance, which accounted for 11% of total federal spending in 2020 (Hahn et al., 2021).

13 We rely on Urban Institute’s “State-by-State Spending on Kids Dataset”, which “provides a comprehensive accounting of public spending on children from 1997 through 2016”. Urban Institute draws from US Census Bureau’s Annual Survey of State and Local Government Finances for its database. The values are adjusted for inflation and expressed in 2016 real dollars. For details on database construction see: https://datacatalog.urban.org/dataset/state-state-spending-kids-dataset.

14 Childcare and education expenses is one of the categories estimated by the U.S. Department of Agriculture reports and includes “day care tuition and supplies; babysitting; and elementary and high school tuition, books, fees, and supplies” (Lino et al., 2017).

15 Similarly, Zelizer (1985) in her *Pricing the Priceless Child* analysis drew her evidence mostly from historical legal cases and archives of newspapers and magazines, not parent interviews, to get at the cultural meaning and the social value of children. Moreover, Zelizer noted that the focus on a priceless, not economically useful, child started with middle-class children in mind and only later prevailed also among the working class ones.

16 Personal communication over email with Viviana Zelizer by the first author, on October 9, 2022.
